# Associations between perceived quantitative work demands at different organisational levels and pain and sickness absence in eldercare workers: a multi-level longitudinal analysis

**DOI:** 10.1007/s00420-022-01850-y

**Published:** 2022-04-20

**Authors:** Matthew L. Stevens, Kristina Karstad, Svend Erik Mathiassen, Leticia Bergamin Januario, Reiner Rugulies, David M. Hallman, Andreas Holtermann

**Affiliations:** 1 The National Research Centre for the Working Environment, Lersø Parkallé 105, 2100 København Ø, Denmark; 2 Department of Occupational Health Sciences and Psychology, Centre for Musculoskeletal Research, University of Gävle, Gävle, Sweden; 3 Department of Public Health, University of Copenhagen, Copenhagen, Denmark; 4 Department of Psychology, University of Copenhagen, Copenhagen, Denmark; 5 Department of Sports Science and Clinical Biomechanics, University of Southern Denmark, Odense, Denmark

**Keywords:** Job demands, Low back pain, Sickness leave, Aged care, Eldercare, Mixed-effects modelling

## Abstract

**Purpose:**

Eldercare work is characterised by high quantitative work demands and high occurrence of musculoskeletal pain and sickness absence. Our aim was to investigate the association between quantitative demands aggregated at the different organizational levels of eldercare and low back pain (LBP) and sickness absence due to pain among workers.

**Methods:**

This study was conducted in 527 eldercare workers from 105 wards across 20 nursing homes in Denmark. We collected workers’ perceived quantitative demands at baseline and workers’ LBP and sickness absence repeatedly over the following year. We aggregated worker-level quantitative demands to the ward and nursing home-levels, and used mixed-effects regression models to investigate the associations between quantitative demands at different organizational levels and LBP and sickness absence over 1 year.

**Results:**

Across all models, increased quantitative demands (0–100 scale) at the worker-level was associated with an increased likelihood (OR 1.02) and intensity of LBP (*β* = 0.01). We did not identify any associations between quantitative demands at the ward-level and either of our outcomes. Across all models, increased quantitative demands at the nursing home-level was associated with increased days with sickness absence due to pain (*β* = 0.03 to 0.06).

**Conclusion:**

In eldercare, workers’ perceived quantitative demands are associated with the presence and intensity of LBP. Further, quantitative demands across the overall nursing home-level are associated with sickness absence due to pain among eldercare workers. These results are of relevance to developing organisational interventions targeting quantitative demands to reduce sickness absence in eldercare.

**Supplementary Information:**

The online version contains supplementary material available at 10.1007/s00420-022-01850-y.

## Introduction

The European Pillar for Social Rights recognises quality long-term care services as one of the core principles for society (European Commission 2017). Additionally, the number of Europeans aged 80 + is expected to more than double by 2070 (Spasova et al. 2018), meaning that the demand for elderly care will increase. This increased demand is being accompanied by rising retirement ages, expecting eldercare workers to be able to work to older ages (European Commission 2012). These factors combined add strain to an occupational sector already dealing with poor working conditions and high staff turnover (EU Skills Panorama 2014). To strengthen this important sector, a proper understanding of occupational factors influencing working life and early retirement among eldercare workers is key (European Commission 2012; Clausen et al. 2014; Roen et al. 2018; Spasova et al. 2018).

One of the primary reasons for sickness absence and subsequent early retirement among workers is low back pain (LBP) (Hartvigsen et al. 2018). Such pain tends to occur episodically, with periods of pain that occur, and generally subside to a low or pain-free level before flaring up again (Suri et al. 2012; Hancock et al. 2015). These episodes can be influenced by physical (in eldercare, e.g., manual handling of residents), and cognitive (e.g., emotionally demanding residents, competing priorities/distraction) work (Steffens et al. 2015; Stevens et al. 2016). To capture this combination of stressful physical and mental factors, researchers commonly ask workers about their perceived quantitative work demands, referring to the work required to be performed relative to the time available to conduct the work (Kristensen et al. 2004). Perceived quantitative work demands, hereafter referred to as ‘quantitative demands’, have been associated with workers’ mental and physical health, long-term sickness absence and subsequent early retirement (Jansen et al. 2004; Schütte et al. 2014; Slany et al. 2014; Freimann et al. 2016).

Although high quantitative demands are commonly associated with detrimental health outcomes, most studies are limited by having only investigated the associations for individual workers without consideration for the organizational structure within which the work is conducted. For example, in eldercare, the workers are employed in wards, which are sub-units compiled within nursing homes. This information is of importance because it provides the context within which a worker conducts their work. In order to move prevention measures beyond initiatives directed towards the individual worker, we need to know how quantitative demands at different levels of an organisation (in eldercare these being the worker, ward and nursing home-levels) are associated with important health outcomes, such as LBP and sickness absence.

Therefore, our aim was to investigate the association between quantitative demands measured at the individual worker-level and aggregated at the ward and nursing home-level, and LBP and sickness absence over a 12-month period among eldercare workers.

## Methods

This study used data from the Danish Observational Study of Eldercare work and musculoskeletal disorderS (DOSES) (Karstad et al. 2018)—a Danish cohort of workers in elderly care collected from September 2013 to January 2016. Ethical approval for DOSES was provided by the Danish Data Protection Agency and the Ethics Committee for the regional capital of Denmark (H-4-2013-028). The full details of DOSES have been previously published (Karstad et al. 2018).

### Study participants

We purposively selected and invited 83 nursing homes located in Zealand and the capital region of Denmark to participate in the study. The aim of the selection was to include nursing homes of various sizes and care models. Of the 83 nursing homes invited, 20 nursing homes (127 wards) agreed to participate and were subsequently included. After a nursing home agreed to participate, we distributed written information about the aim and activities of the research to all employees and arranged an information meeting at the nursing home to inform employees about the study and invite them to participate. Participants in the study were eldercare workers from 18 to 65 years of age, employed in nursing homes more than 15 h/week on day and evening shifts and spent a minimum of 25% of their working time on tasks related to direct care of residents. This ensured that our population of workers regularly contributed to caring activities, primarily worked in eldercare and worked enough to be affected by it.

### Data collection

Exposure, covariate and contextual variables were measured at baseline while the outcome variables were collected over the 1-year period immediately following baseline data collection. Baseline data collection for nursing home managers and team managers (responsible for the wards) consisted of a web-based questionnaire about formal and informal organizational structures at the nursing home and wards. Baseline data collection for workers included a structured self-administered questionnaire, which was filled in during a health check at the work place with research personnel present to help if necessary. Follow-up data were collected by having participating workers answer short-message service (SMS) prompts sent to their mobile phones. All data were collected electronically with impossible values unable to be entered. Data were also systematically cross-checked for errors by researchers and data managers.

### Exposure

Quantitative Demands were measured using two items from the Copenhagen Psychosocial Questionnaire (COPSOQ) II (Pejtersen et al. 2010): ‘Do you get behind with your work?’ and ‘Do you have enough time for your work tasks?’ (reverse coded). Response options were as follows: ‘always’, ‘often’, ‘sometimes’, ‘rarely’ or ‘never/almost never’. We converted the responses to each of the two items for each worker to a five-point scale (0, 25, 50, 75, 100), with higher values indicating greater quantitative demands, and then averaged the two ratings. Next, we aggregated these worker-level quantitative demands, first at the ward-level and then at the nursing home-level.

### Outcomes

For each level of quantitative demands (worker, ward and nursing home), we examined the following three outcomes: the likelihood of LBP over the previous 4 weeks (yes/no), the intensity of LBP over the previous 4 weeks in those with pain (0–10 scale) and the number of days with sickness absence due to pain in the previous 12 weeks (0–84 days). Every 4 weeks (for 1 year), workers were sent an SMS asking their number of days with LBP in the previous 4 weeks (0–28 days). To obtain our measure of presence of LBP we then dichotomised this response into either yes or no. If workers responded that they had at least 1 day with LBP, they were then sent an SMS asking them to rate their maximum pain intensity over the previous 4 weeks. Sickness absence was obtained via the same method. Every 12 weeks workers were sent an SMS asking how many days they had been absent from work in the past 12 weeks (0–84 days). To obtain sickness absence due to pain, workers who responded with any sickness absence were then sent a second SMS asking how many days of that sickness absence was due to either their LBP or neck/shoulder pain. If workers did not respond to the SMS they were called and the information was collected over the phone.

### Covariates

We collected information on age, sex, body mass index (BMI), emotional demands at work and the resident-staff ratio. Age, sex and emotional demands were self-reported by workers, with emotional demands collected using all four items from the COPSOQ II (Pejtersen et al. 2010) (‘Does your work put you in emotionally disturbing situations?’, ‘Do you have to relate to other people’s personal problems as part of your work?’, ‘Is your work emotionally demanding?’, ‘Do you get emotionally involved in your work?’). Each item was rated on a 5-point Likert scale with response option: ‘always’, ‘often’, ‘sometimes’, ‘rarely’ or ‘never/almost never’. For inclusion in the analysis we took the mean of all 4 items and converted it to a 0–100 scale (Pejtersen et al. 2010). We calculated BMI from measurements of height and weight conducted by trained researchers at baseline. Weight was measured to the nearest 0.1 kg/0.1% by trained research personnel using the Tanita (model BC418 MA) bio-impedance segmental body composition analyser. The resident-staff ratio was calculated from information obtained from the ward managers. This was the regular number of residents in that ward divided by the regular number of workers on that shift (i.e., day or evening shift). We then assigned this value to workers depending upon whether they worked day shifts, evening shifts, or day and evening shifts.

### Contextual factors

Other presented variables are the workers’ job classification, the type of ward where they worked and workers’ perceived general health. Workers’ employment/job was divided into the following three categories: ‘care helpers’ (who had 14 months of training in care provision), ‘care aides’ (who had completed an additional 6 months of training) and ‘nurses or other health professionals’. The extra education undertaken by the care aides (as opposed to care helpers) allows them to independently handle medications (within limits) while care helpers do not. In general, care aides also have more responsibility, coordinating different tasks and information in and between teams, while care helpers are limited to providing care and practical help to the resident. Wards were divided into four types—somatic, dementia, temporary rehabilitation and psychiatric. Perceived general health was obtained from workers using a single item from the SF-36 (Brazier et al. 1992) ‘In general, would you say your health is:’ with five possible responses—‘excellent’, ‘very good’, ‘good’, ‘fair’ or ‘poor’.

### Statistical analysis

To investigate the relationship between quantitative demands and our outcomes (LBP and sickness absence), we developed four multi-level regression models for each outcome with each model having two versions, i.e. an unadjusted and an adjusted version that included age, sex, BMI, emotional work demands and the resident/staff ratio. To take into account clustering at four different levels (reporting time-point within workers, workers within wards, wards within nursing homes and nursing homes) all models used a mixed-effects structure that included the individual, ward and nursing home as random intercepts. Model 1 was a worker-level analysis. As such, model 1 utilised worker-level data of quantitative demands (i.e., no aggregation). Model 2 was a ward-level analysis that used quantitative demands data aggregated to the ward-level. Model 3 was a nursing home-level analysis and used data aggregated to the nursing home-level. In other words, for Models 2 and 3 we took a group-based exposure assessment approach, where workers were assigned the average exposure (quantitative demands) for the ward (Model 2) or nursing home (Model 3) where they worked, rather than getting their own personal exposure. The outcome measures were always on the individual-level. Model 4 was a combined model that included measures of quantitative demands at all levels (i.e., workers personal rating of quantitative demands, a ward-level aggregate measure of quantitative demands and a nursing home-level aggregate of quantitative demands). We conducted models 1–3 to investigate quantitative demands at different organisational levels. We conducted Model 4 to assess whether these different organisational levels act independently of each other.

The outcome determined the type of regression model used. This was decided based upon the characteristics of the data and the Akaike information criterion (AIC) values. In the models presented, we used binomial, Gaussian and negative-binomial regression for the presence of LBP, intensity of LBP and sickness absence models, respectively. In the adjusted versions of each model, we adjusted for age, sex, BMI, emotional demands and the resident-staff ratio. Participants with missing outcome values (e.g., missing data at specific timepoints) remained in the analyses and contributed with the values they had. Participants with missing values vital to a particular analysis (e.g., a covariate in the adjusted analyses) were removed from that analysis. All analyses were conducted in R v4.0.2 (R Core Team 2018) and RStudio (RStudio Team 2016) with packages glmmTMB (Magnusson et al. 2020), broom.mixed (Bolker et al. 2020), DHARMa (Hartig 2020), effects (Fox et al. 2020) and the tidyverse suite of packages (Wickham et al. 2019).

To test the robustness of our results and to gain a greater understanding of the role of time in the relationships investigated, we conducted two sensitivity analyses. The first was to assess sickness absence due to pain only in those with pain. For this analysis pain was defined as a pain intensity ≥ 3/10 for more than 1 day. This is in line with recommendations for what might be considered ‘important’ pain (Stanton et al. 2011). The second sensitivity analysis was to examine the impact of time on the effect of quantitative demands on our outcomes over 1 year and in doing so take into account the relationship between our outcomes at different time-points (e.g., baseline pain on later pain time-points). To do this, we added time (and its interaction with quantitative demands) to the unadjusted and adjusted versions of models 1 to 3.

## Results

We included 527 eldercare workers in this analysis, employed at 105 wards in the 20 participating nursing homes. Eldercare workers were generally middle-aged [mean 45.5 years, standard deviation (SD) 10.9] and nearly all were females (95.3%). Most were either care aides (46.5%) or care helpers (43.4%) and worked in somatic wards (75.1%). The average exposure to Quantitative demands (scale 0–100) at the worker, ward and nursing home-levels were 44.4 (SD 20.2), 44.6 (SD 13.1) and 45.3 (SD 8.0), respectively. Roughly two-thirds (66.9%) of workers had LBP at baseline with an average LBP intensity (among those with LBP) of 5.1 (0–10 scale; SD 2.3). Over the baseline period (collected at 4 weeks) workers had, on average, around half a day of sickness absence due to pain (mean 0.4 (SD 1.9); median 0). During follow-up, LBP and sickness absence were similar as baseline at the group level. Full details are provided in Table 1.

**Table 1:** Demographics information for 527 Danish eldercare workers

	Mean (SD), *n* (%) or median (IQR)
Age (years)	45.5 (SD 10.9)
Sex (f)	502 (95.3%)
BMI (kg/m^2^; *n* = 491)	26.5 (SD 5.3)
Perceived Health (*n* = 518)	
Excellent	16 (3.1%)
Very good	140 (27.0%)
Good	280 (54.1%)
Not so good	75 (14.5%)
Poor	7 (1.4%)
Job type (*n* = 523)	
Care Aide	243 (46.5%)
Care Helper	227 (43.4%)
Nurse or other Health Professional	53 (10.1%)
Ward type	
Somatic	396 (75.1%)
Dementia	107 (20.3%)
Temporary rehab	15 (2.8%)
Psychiatric	9 (1.7%)
Perceived Quantitative Demands at baseline (0–100 scale)	44.5 (SD 20.2)
Presence of LBP at baseline (*n* = 505)	338 (66.9%)
LBP intensity among those with pain at baseline (0–10 scale) (*n* = 338)	5.1 (SD 2.3)
Proportion with Sickness Absence due to pain at 4-weeks follow-up (*n* = 468)	46 (9.8%)
Sickness Absence due to pain at 4-weeks (0–84 days) (*n* = 468)*	0.4 (SD 1.9)
Follow-up data	
Presence of LBP across all time-points (*n* = 6339)	4203 (66.3%)
LBP intensity among those with pain across all time-points (0–10 scale) (*n* = 4203)	5.2 (SD 2.3)
Proportion across all time-points with Sickness Absence due to pain (*n* = 2207)	271 (12.3%)
Sickness Absence due to pain across all time-points (0–84 days) (*n* = 271)*	0.9 (SD 5.5)

*SD* standard deviation, *IQR* inter-quartile range, *BMI* body mass index, *LBP* low back pain

*Median (IQR) at both baseline (4 weeks) and follow-up was 0 (0 to 0)

### Quantitative demands vs. presence of LBP

In all models, increased quantitative demands were associated with an increased likelihood of LBP among eldercare workers. However, the association was only significant at the worker level (OR across all worker-level models 1.02 [lower CI = 1.00; upper CI = 1.03 to 1.04]; Table 2). This effect corresponds to a 10-point increase in quantitative demands at the worker-level (on a 0–100 scale) being associated with a 20% increase in the odds of having LBP during any 4-week period, over the following year. This association remained consistent across unadjusted and adjusted models and in the combined models. In the ward and nursing home-level analyses, quantitative demands showed a similar (or stronger) association with the presence of LBP, but the associations were not statistically significant.

**Table 2:** Association between perceived quantitative work demands (0–100 scale) and the presence of low back pain (yes/no) among Danish eldercare workers

	Presence of low back pain		
	*R*^2^_m_	Odds ratio	*p* value
Unadjusted			
Worker level	0.01	**1.02 [1.00; 1.04]**	**0.012**
Ward level	< 0.01	1.02 [0.99; 1.06]	0.128
Nursing home level	< 0.01	1.06 [0.99; 1.12]	0.090
Combined*	0.02		
Worker		**1.02 [1.00; 1.03]**	**0.036**
Ward		0.99 [0.95; 1.03]	0.713
Nursing home		1.04 [0.97; 1.12]	0.262
Adjusted**			
Worker level	0.05	**1.02 [1.00; 1.04]**	**0.017**
Ward level	0.04	1.01 [0.98; 1.05]	0.499
Nursing home level	0.05	1.04 [0.98; 1.11]	0.200
Combined	0.06		
Worker		**1.02 [1.00; 1.04]**	**0.022**
Ward		0.98 [0.94; 1.02]	0.348
Nursing home		1.04 [0.96; 1.12]	0.313

Bold values indicate statistical significance (*p* < 0.05)

*R*^*2*^_*m*_ marginal *R*^2^: the variance explained by the fixed effects in the model only

*The combined model includes all three quantitative demands variables (i.e., worker-level, ward-level and nursing home-level) in the same model

**Models have been adjusted for age, sex, BMI, emotional work demands and the resident/staff ratio

### Quantitative demands vs. intensity of LBP

Results for the intensity of LBP were similar to those for the presence of LBP. When considering the intensity of LBP, higher quantitative demands were weakly associated with higher LBP intensity (Table 3). However, again this was only significant at the worker level (β across all worker level models = 0.01 [lower CI = 0.00 to 0.01; upper CI = 0.02]), meaning that a 10-point increase in quantitative demands at the worker-level (0–100 scale) was significantly associated with an increase of 0.1 in LBP intensity (0–10 scale) across all time-points over the following year. This association remained consistent across unadjusted and adjusted models and in the combined models. In the ward and nursing home-level analyses, quantitative demands still showed a similar association with the presence of LBP; however, these associations were not statistically significant.

**Table 3:** Association between perceived quantitative work demands (0–100 scale) and the intensity of low back pain (0–10 scale) among Danish eldercare workers with pain

	Intensity of low back pain in those with low back pain		
	*R*^2^_m_	*β*	*p* value
Unadjusted			
Worker level	0.01	**0.01 [0.01; 0.02]**	**0.001**
Ward level	< 0.01	0.01 [− 0.00; 0.03]	0.069
Nursing home level	< 0.01	0.02 [− 0.01; 0.05]	0.112
Combined*	0.02		
Worker		**0.01 [0.00; 0.02]**	**0.007**
Ward		− 0.00 [− 0.02; 0.02]	0.902
Nursing home		0.01 [− 0.02; 0.05]	0.411
Adjusted**			
Worker level	0.03	**0.01 [0.00; 0.02]**	**0.012**
Ward level	0.02	0.01 [− 0.00; 0.03]	0.140
Nursing home level	0.02	0.03 [− 0.00; 0.06]	0.101
Combined	0.03		
Worker		**0.01 [0.00; 0.02]**	**0.045**
Ward		− 0.00 [− 0.02; 0.02]	0.877
Nursing home		0.02 [− 0.02; 0.05]	0.362

*R*^*2*^_*m*_ marginal *R*^2^: the variance explained by the fixed effects in the model only

*The combined model includes all three quantitative demands variables (i.e., worker-level, ward-level and nursing home-level) in the same model

**Models have been adjusted for age, sex, BMI, emotional work demands and the resident/staff ratio

### Quantitative demands vs. sickness absence due to pain

Increased quantitative demands were associated with an increased in sickness absence due to pain; however, this association only occurred in the nursing home-level analysis (Table 4). This association was statistically significant in the univariate, unadjusted analysis (*β* = 0.04 [lower CI = 0.00; upper CI = 0.08]), indicating that a 10 point increase in quantitative demands at the nursing homelevel was associated with a 0.4 increase in the number of days with sickness absence due to pain per worker per 12 week period, over the following year. However, the statistical significance of this effect was lost in the adjusted analysis (univariate, adjusted analysis: *β* = 0.03 [lower CI = − 0.01; upper CI = 0.08]) but was present (and strengthened) in both combined models (combined, unadjusted analysis: *β* = 0.07 [lower CI = 0.02; upper CI = 0.12]; combined, adjusted analysis: *β* = 0.06 [lower CI = 0.00; upper CI = 0.12]).

**Table 4:** Association between perceived quantitative work demands (0–100 scale) and days with sickness absence due to pain (0–84 days) among Danish eldercare workers

	Sickness Absence due to Pain		
	*R*^2^_m_	*β*	*p* value
Unadjusted			
Worker level	< 0.01	0.00 [− 0.01; 0.02]	0.561
Ward level	< 0.01	0.01 [− 0.02; 0.03]	0.487
Nursing home level	0.02	**0.04 [0.00; 0.08]**	**0.029**
Combined*	0.04		
Worker		0.00 [− 0.01; 0.02]	0.735
Ward		− 0.02 [− 0.06; 0.01]	0.197
Nursing home		**0.07 [0.02; 0.12]**	**0.011**
Adjusted**			
Worker level	0.09	0.00 [− 0.01; 0.02]	0.736
Ward level	0.08	− 0.00 [− 0.03; 0.03]	0.974
Nursing home level	0.10	0.03 [− 0.01; 0.08]	0.123
Combined	0.11		
Worker		0.00 [− 0.01; 0.02]	0.660
Ward		− 0.03 [− 0.07; 0.01]	0.144
Nursing home		**0.06 [0.00; 0.12]**	**0.037**

*R*^*2*^_*m*_ marginal *R*^2^: the variance explained by the fixed effects in the model only

*The combined model includes all three quantitative demands variables (i.e., worker-level, ward-level and nursing home-level) in the same model

**Models have been adjusted for age, sex, BMI, emotional work demands and the resident/staff ratio

### Sensitivity analyses

Our sensitivity analyses investigating the effect of quantitative demands on sickness absence due to pain only in those with pain at baseline showed almost identical results to the primary analysis. Full details are provided in the online Appendix (Table S1). Including the interaction between time and quantitative demands showed significant effects for all of our outcomes (Table S2). As time progressed over our 1-year follow-up period, the association between quantitative demands at baseline and whether or not a worker had LBP (Figs. S1–6) decreased, but the association between quantitative demands and pain intensity increased (Figs. S7–12). As time progressed over 1 year, the association between quantitative demands and sickness absence due to pain increased (Figs. S13–14).

## Discussion

This study found that increased worker-level quantitative demands were associated with increased LBP (both the likelihood of having LBP and its intensity) and that increased quantitative demands aggregated at the nursing home-level were associated with an increased risk of sickness absence due to pain. We did not find an association between quantitative demands aggregated at the ward-level and either LBP or sickness absence.

The unique strength of this paper was the use of aggregates of quantitative demands at different organisational levels of eldercare work. Other strengths include the longitudinal repeated collection of LBP and sickness absence data. Furthermore, any potential issue of reverse causation (where our exposure, i.e. quantitative demands, is caused by the outcome) is reduced by our use of aggregated quantitative demands, longitudinal data over 1 year, and the inclusion of time in the sensitivity analyses which adjusts for previous responses.

A limitation of our study is that quantitative demands are still a self-reported measure and that sickness absence was also based on self-report, which might be influenced by recall bias over the 3-month response window. Previous research on quantitative demands has been criticised because of its self-reported nature, which makes common methods bias (where the measurement method creates variance, rather than the constructs intended to be measured) a potential issue (Podsakoff et al. 2003). However, the use of aggregated quantitative demands at the ward and nursing home levels reduces common methods bias considerably (Podsakoff et al. 2003; Croon and Van Veldhoven 2007).

Our study found that increased worker-level quantitative demands were associated with an increased presence and intensity of LBP in eldercare workers but not with sickness absence. Our findings for LBP are in agreement with the previous literature that suggests across different work groups that increased (worker-level) quantitative demands are associated with increased pain (Jansen et al. 2004; Freimann et al. 2016; Peters et al. 2018). As such, it seems that worker-level quantitative demands are consistently related to the subjective experience of the LBP. This may be because of a true effect of quantitative demands on LBP or because of factors such as common method bias. Unfortunately, the sensitivity analyses (interaction with time) do not provide a clear answer. Including the interaction with time in the model showed that the association between quantitative demands and the risk of having LBP decreased over time which could suggest regression to the mean. However, these analyses also showed that the association between quantitative demands and the intensity of LBP increased over time. As such, our results provide no clear answers when considering the relationship between quantitative demands among workers and changes in LBP.

Our analyses at the ward and nursing home-levels showed no associations between ward-level quantitative demands and any of our outcomes, and that nursing home-level quantitative demands was only significantly associated with sickness absence. This implies that LBP is mainly determined by differences in perceived quantitative demands between workers within a ward, rather than differences between wards (within nursing homes) or nursing homes. Our lack of significant findings at ward or nursing home-levels leave us concerned that the significant worker-level association might be biased by reporting bias/reverse causation. However, although the associations between nursing home-level quantitative demands and LBP were not significant, the effect sizes of these associations were either the same or stronger. Furthermore, the lower variation in quantitative demands at the nursing home-level (suggesting that quantitative demands between organisations are similar) may make it difficult to find associations at this level. As such, this suggests no clear picture as to the nature of the relationship between quantitative demands at the ward and nursing home-levels and LBP. Regarding sickness absence, some studies support the idea that quantitative demands is related to sickness absence (Slany et al. 2014; Peters et al. 2018), while others disagree (Hoogendoorn et al. 2002; Clausen et al. 2012; Thorsen et al. 2013), and some show inconsistent relationships (Otsuka et al. 2007). Our study provides a possible explanation for the inconsistent relationships shown in the literature, suggesting that quantitative demands at the nursing home level is important for sickness absence due to pain. This may be because in eldercare workplaces with low quantitative demands, the overburdened worker has the potential to unload some of their burden onto their colleagues and are thus able to continue working whilst they recover. Other reasons include the differences in resources (e.g., some nursing homes would be based in lower social-economic status areas) or cultures (e.g., around when to take sick leave) that likely vary across different nursing homes (Jensen et al. 2011).

The main implication of our findings is to consider the potential importance of targeting when designing interventions to reduce excessive quantitative demands in eldercare work, i.e., whether to prioritize high quantitative demands and greater short-term productivity against lower quantitative demands, better health and greater long-term productivity. Since quantitative demands aggregated at the nursing home-level (but not worker-level or ward-level) were associated with sickness absence, this suggests that interventions to reduce sickness absence in the eldercare should target nursing home-level quantitative demands. In other words, the interventions should (more or less) affect all workers as opposed to trying to target specific ‘at risk’ wards or workers (e.g., organizational interventions such as implementing common routines/tools for organizing and planning work to optimize demands). Such upper organizational-level interventions are also generally simpler and easier to implement as they do not require explicit targeting and tailoring. Our results suggest that a ten-point reduction in quantitative demands (0–100 scale) at the nursing home-level could result in a decrease in sickness absence of roughly half a day per quarter (i.e., roughly 2 days per person over the following year). As such, interventions to reduce quantitative demands in the workforce may result in substantial savings for industry. Even though our models, in being linear, predict that pain and sickness absence will reduce if demands are decreased, we emphasize that very low demands are not desirable either; the optimal job may offer sufficient demands to engage the worker, but not demands to an extent that compromise health.

Future research in this area should investigate if our findings are generalisable across different occupations (e.g., that quantitative demands aggregated at a ‘whole of organisation’-level are associated with sickness absence). Furthermore, in order to build a strong causal argument, RCTs investigating interventions that target quantitative demands in order to reduce sickness absence should investigate whether quantitative demands aggregated to the nursing home-level mediates the effect of the intervention on sickness absence. These mediation studies can also include other important factors (e.g., emotional demands, sleep quality, mental health) (Zhang et al. 2017; Suh and Punnett 2021) and should consider the use of an objective collection of sickness absence to build a full model of health and sickness absence in eldercare workers.

## Conclusion

Our study shows that, in eldercare, workers’ individual perceived quantitative demands are associated with the presence and severity of LBP, but this does not occur when we aggregate the quantitative demands to the ward-level. Moreover, it seems that quantitative demands across the entire workplace is of importance for sickness absence. Thus, it seems that generic organizational interventions aiming to reduce quantitative demands may be suitable to target for reducing sickness absence, but are unlikely to reduce LBP among eldercare workers. If reduction of LBP among workers is the aim, specific, targeted interventions may be more effective than generic interventions at ward or workplace-levels.

## Supplementary Information

Below is the link to the electronic supplementary material.

Supplementary file1 (PDF 1790 kb)

## Data Availability

Data can be made available upon request.
