# Supplementary material for: Associations between perceived quantitative work demands at different organisational levels and pain and sickness absence in eldercare workers: a multi-level longitudinal analysis
Source: Int Arch Occup Environ Health. 2022 Apr 20;95(5):993–1001. doi: 10.1007/s00420-022-01850-y (PMC9203390; doi:10.1007/s00420-022-01850-y)

Associations between Perceived Quantitative Work Demands at Different Organisational Levels and Pain and Sickness Absence in Eldercare Workers: A Multi-level Longitudinal Analysis

Online Appendix

Table S1: Associations between quantitative demands and sickness absence due to pain among Danish workers with pain >= 3/10 for >1 day

	Sickness Absence due to Pain in those with Pain*		
	R2m	B	p-value
**Unadjusted**			
Worker level	<0.01	0.00 [-0.01; 0.01]	0.878
Ward level	<0.01	0.00 [-0.02; 0.03]	0.746
Nursing home level	0.03	**0.04 [0.00; 0.08]**	**0.040**
Combined	0.05		
Worker		0.00 [-0.02; 0.02]	0.971
Ward		-0.03 [-0.06; 0.01]	0.131
Nursing Home		**0.07 [0.02; 0.12]**	**0.007**
**Adjusted**			
Worker level	0.07	0.00 [-0.01; 0.01]	0.997
Ward level	0.07	-0.00 [-0.02; 0.02]	0.936
Nursing home level	0.09	0.03 [-0.01; 0.07]	0.122
Combined	0.12		
Worker		-0.00 [-0.02; 0.01]	0.398
Ward		0.01 [-0.02; 0.03]	0.622
Nursing Home		**0.04 [0.00; 0.07]**	**0.029**
* defined as a pain intensity >=3/10 for more than 1 day			

Table S2: Interaction between quantitative demands and time on various outcomes among Danish workers.

	Presence of LBP		Intensity of LBP		Sickness Absence due to Pain	
	Odds ratio	p-value	B	p-value	B	p-value
**Unadjusted**						
Worker level	**0.999 [0.998; 1.000]**	**0.022**	**0.001 [0.000; 0.002]**	**0.006**	**0.009 [0.001; 0.017]**	**0.020**
	(Figure S1)		(Figure S7)		(Figure S13)	
Ward level	**0.998 [0.996; 1.000]**	**0.016**	**0.001 [0.000; 0.002]**	**0.018**	0.006 [-0.008; 0.020]	0.388
	(Figure S2)		(Figure S8)			
Nursing home level	**0.995 [0.992; 0.999]**	**0.005**	**0.003 [0.001; 0.004]**	**0.002**	0.011 [-0.010; 0.033]	0.306
	(Figure S3)		(Figure S9)			
**Adjusted**						
Worker level	**0.999 [0.997; 1.000]**	**0.009**	**0.001 [0.000; 0.002]**	**0.002**	**0.011 [0.003; 0.019]**	**0.008**
	(Figure S4)		(Figure S10)		(Figure S14)	
Ward level	**0.997 [0.995; 0.999]**	**0.002**	**0.001 [0.000; 0.003]**	**0.018**	0.010 [-0.005; 0.025]	0.173
	(Figure S5)		(Figure S11)			
Nursing home level	**0.994 [0.991; 0.998]**	**0.001**	**0.003 [0.001; 0.004]**	**0.002**	0.017 [-0.007; 0.040]	0.159
	(Figure S6)		(Figure S12)			

Figure S1: Interaction between Quantitative Demands (worker level) and time on the odds of a worker having low back pain – unadjusted model

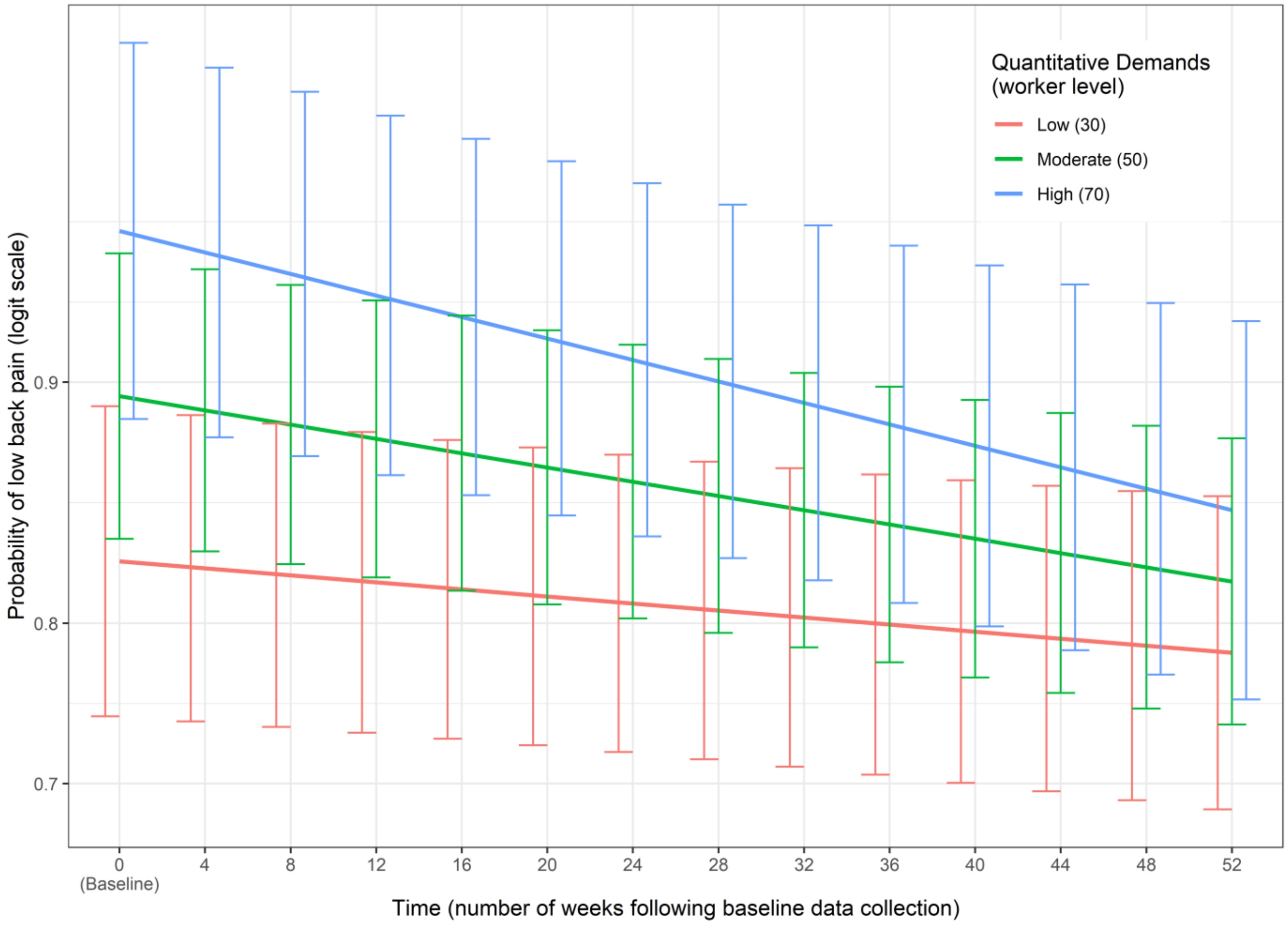

Figure S2: Interaction between Quantitative Demands (worker level) and time on the odds of a worker having low back pain – adjusted for age, sex, BMI emotional demands and staffing ratio

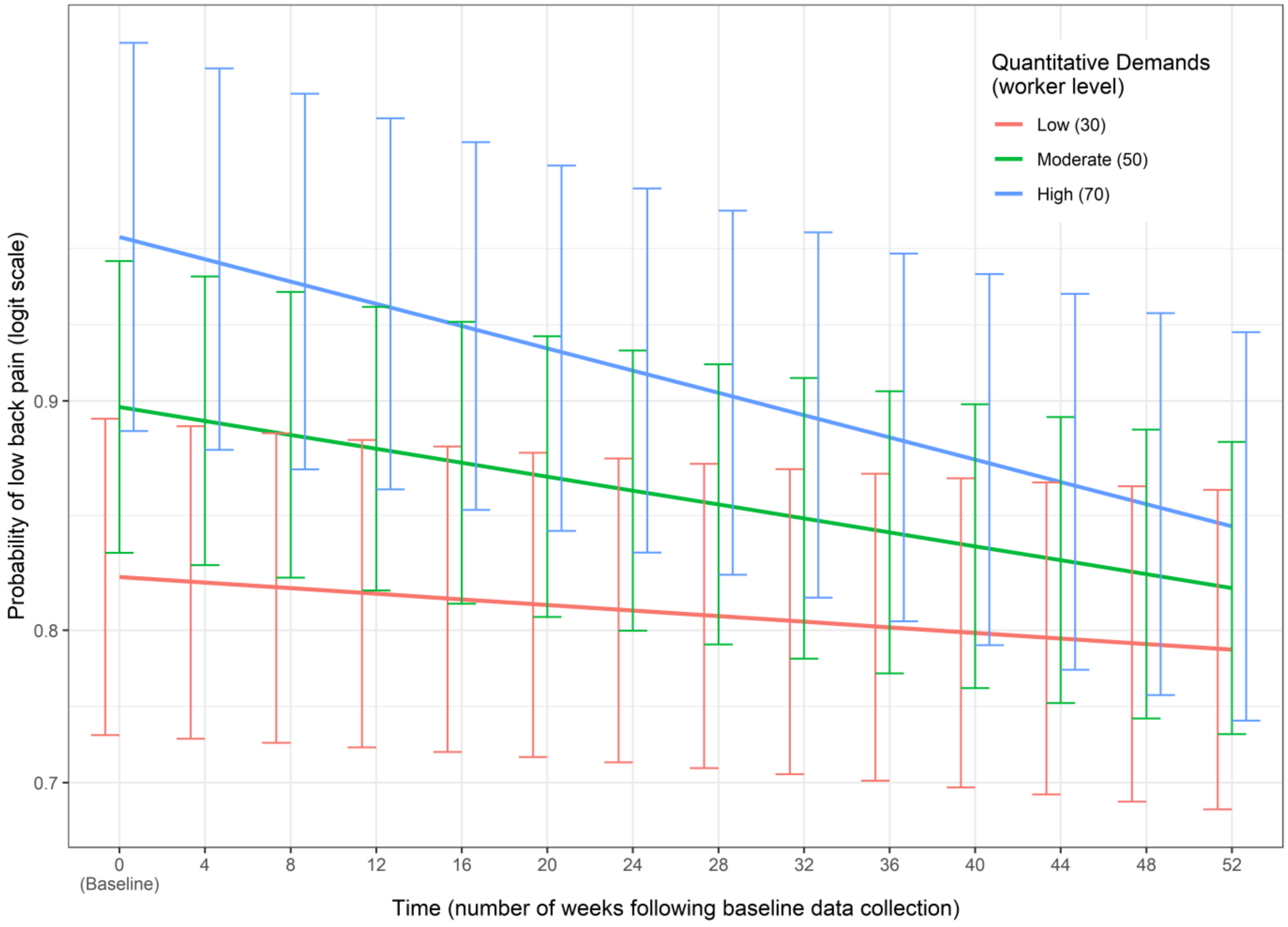

Figure S3: Interaction between Quantitative Demands (ward-level) and time on the odds of a worker having low back pain – unadjusted model

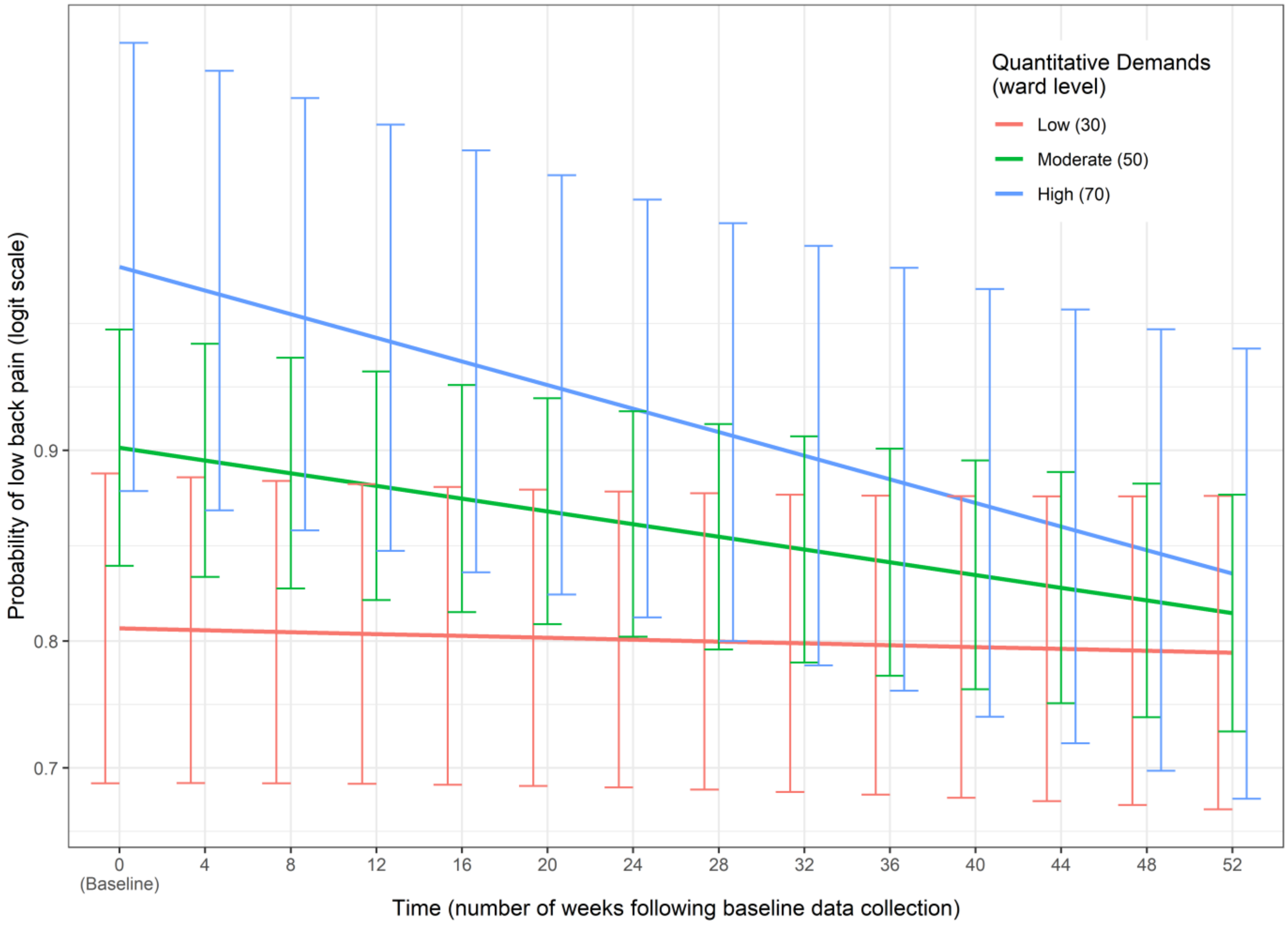

Figure S4: Interaction between Quantitative Demands (ward-level) and time on the odds of a worker having low back pain – adjusted for age, sex, BMI emotional demands and staffing ratio

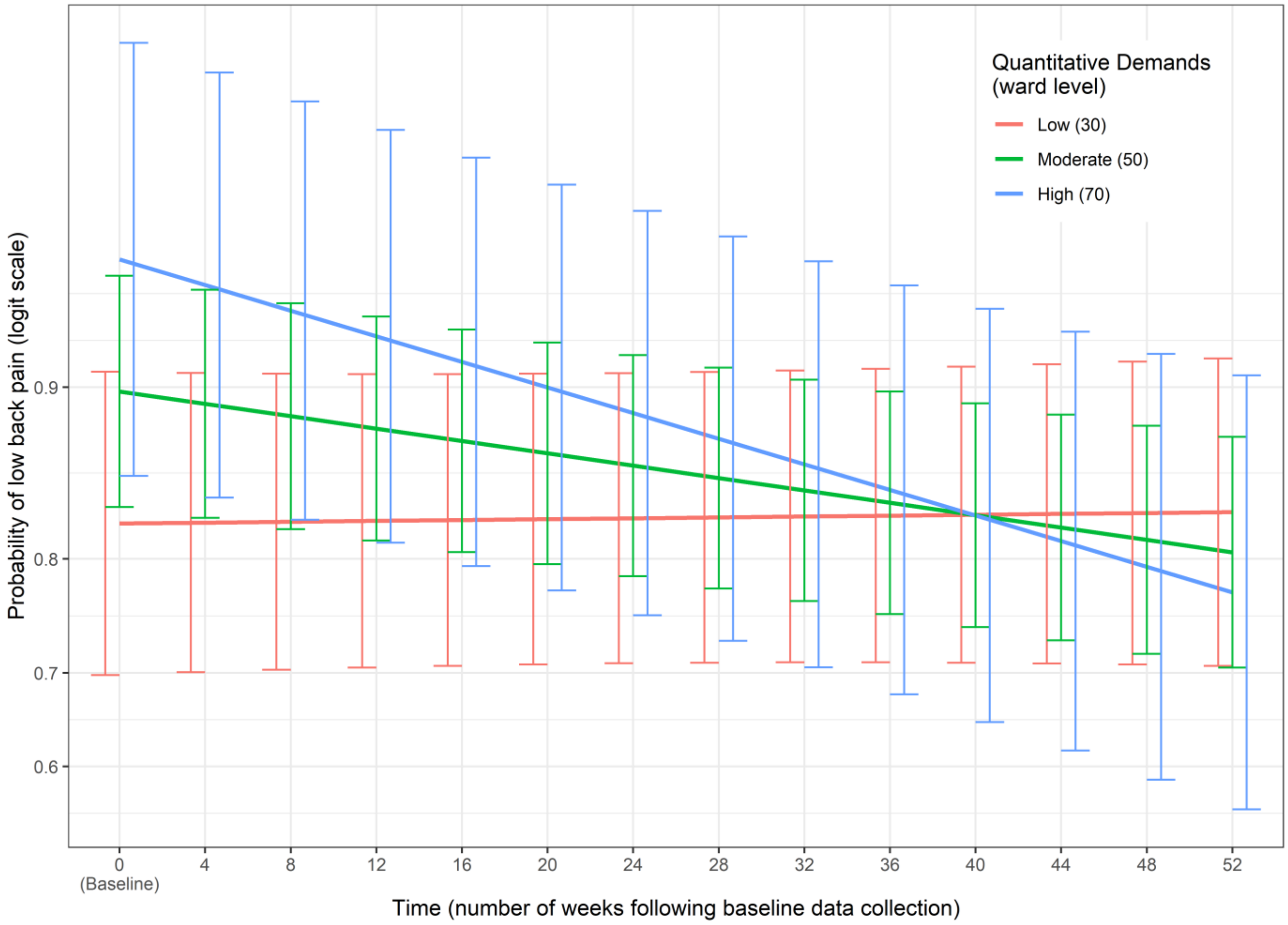

Figure S5: Interaction between Quantitative Demands (nursing home-level) and time on the odds of a worker having low back pain – unadjusted model

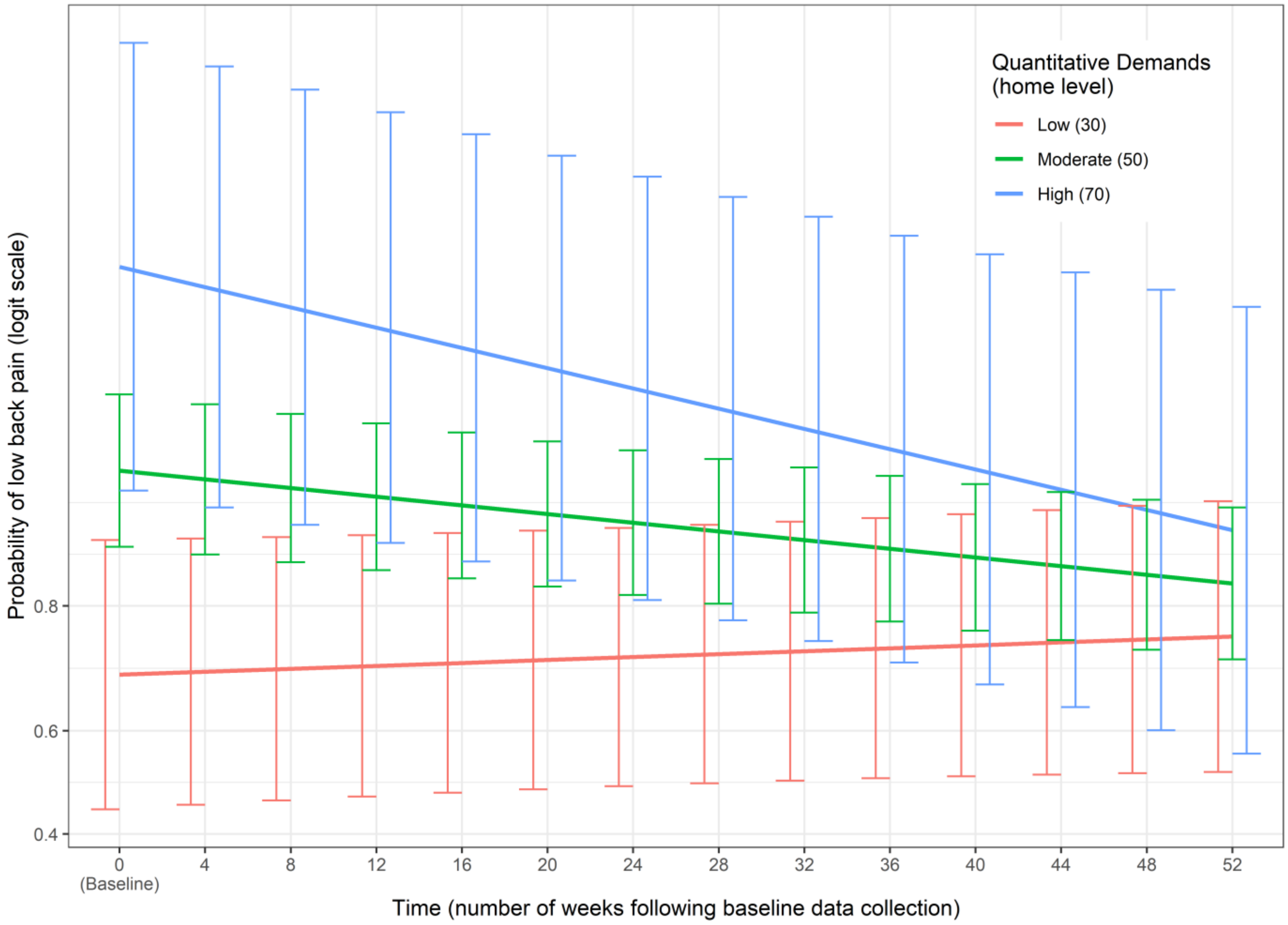

Figure S6: Interaction between Quantitative Demands (nursing home-level) and time on the odds of a worker having low back pain – adjusted for age, sex, BMI emotional demands and staffing ratio

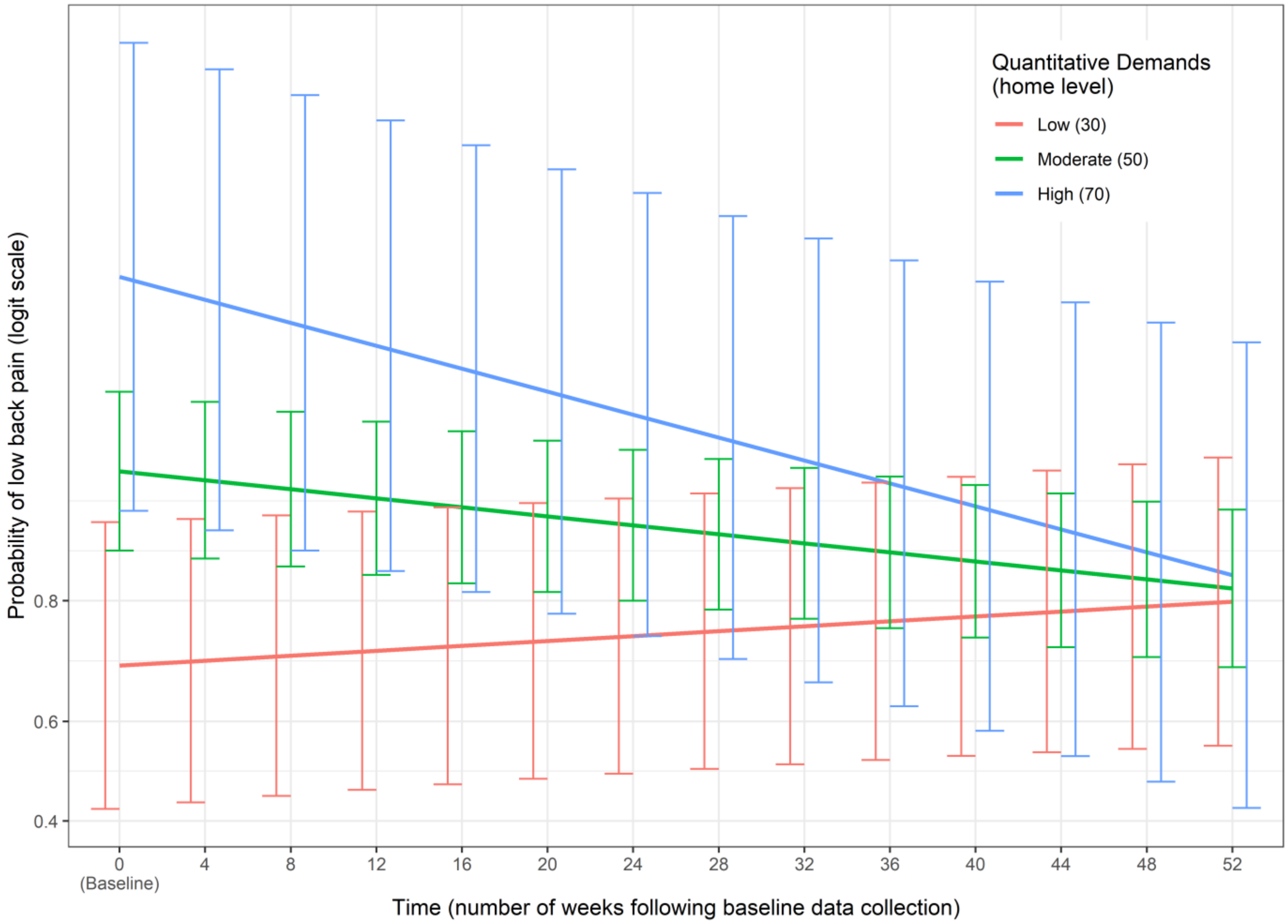

Figure S7: Interaction between Quantitative Demands (worker-level) and time on the intensity of low back pain within workers with low back pain – unadjusted model

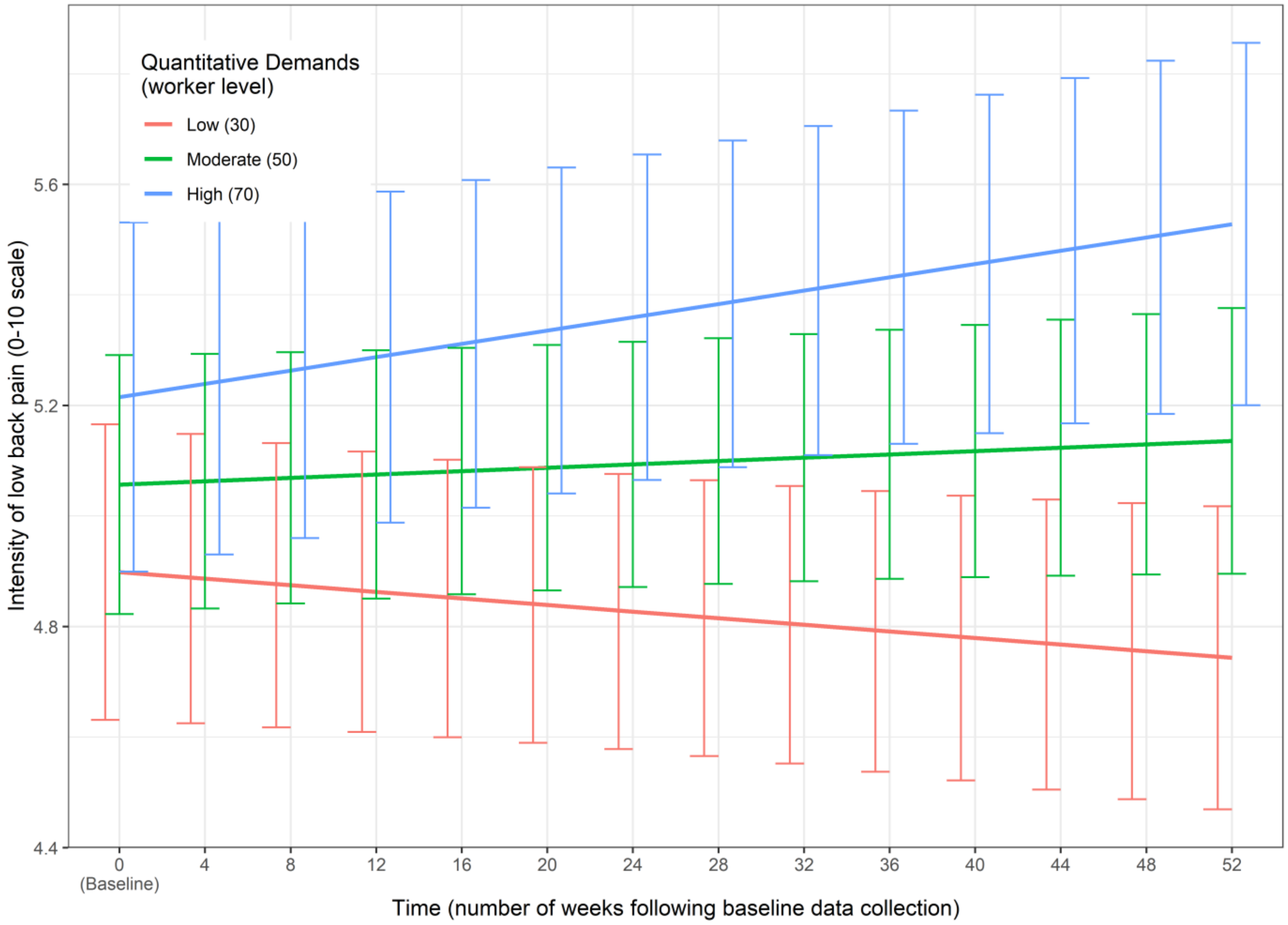

Figure S8: Interaction between Quantitative Demands (worker-level) and time on the intensity of low back pain within workers with low back pain – adjusted for age, sex, BMI emotional demands and staffing ratio

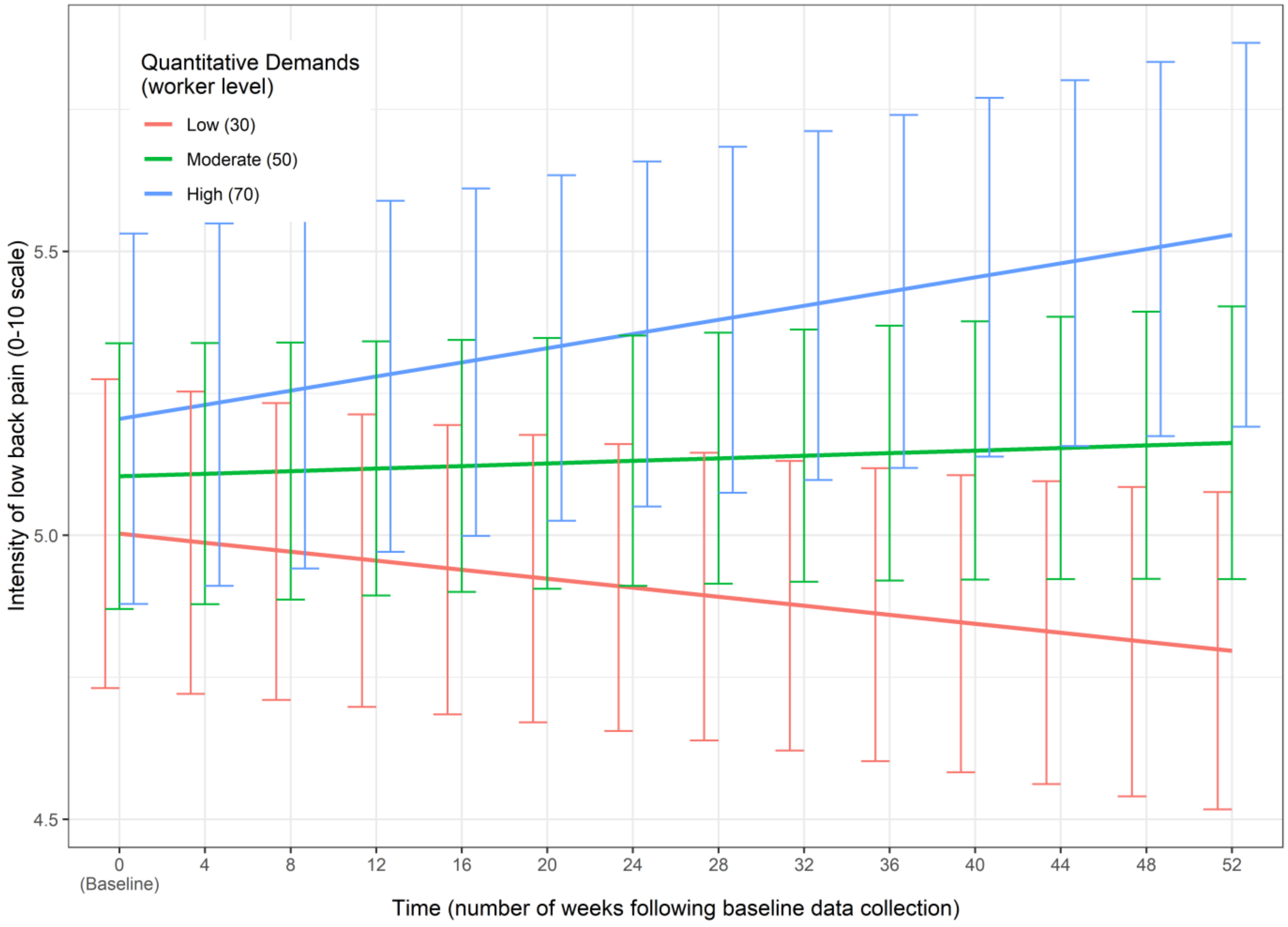

Figure S9: Interaction between Quantitative Demands (ward-level) and time on the intensity of low back pain within workers with low back pain – unadjusted model

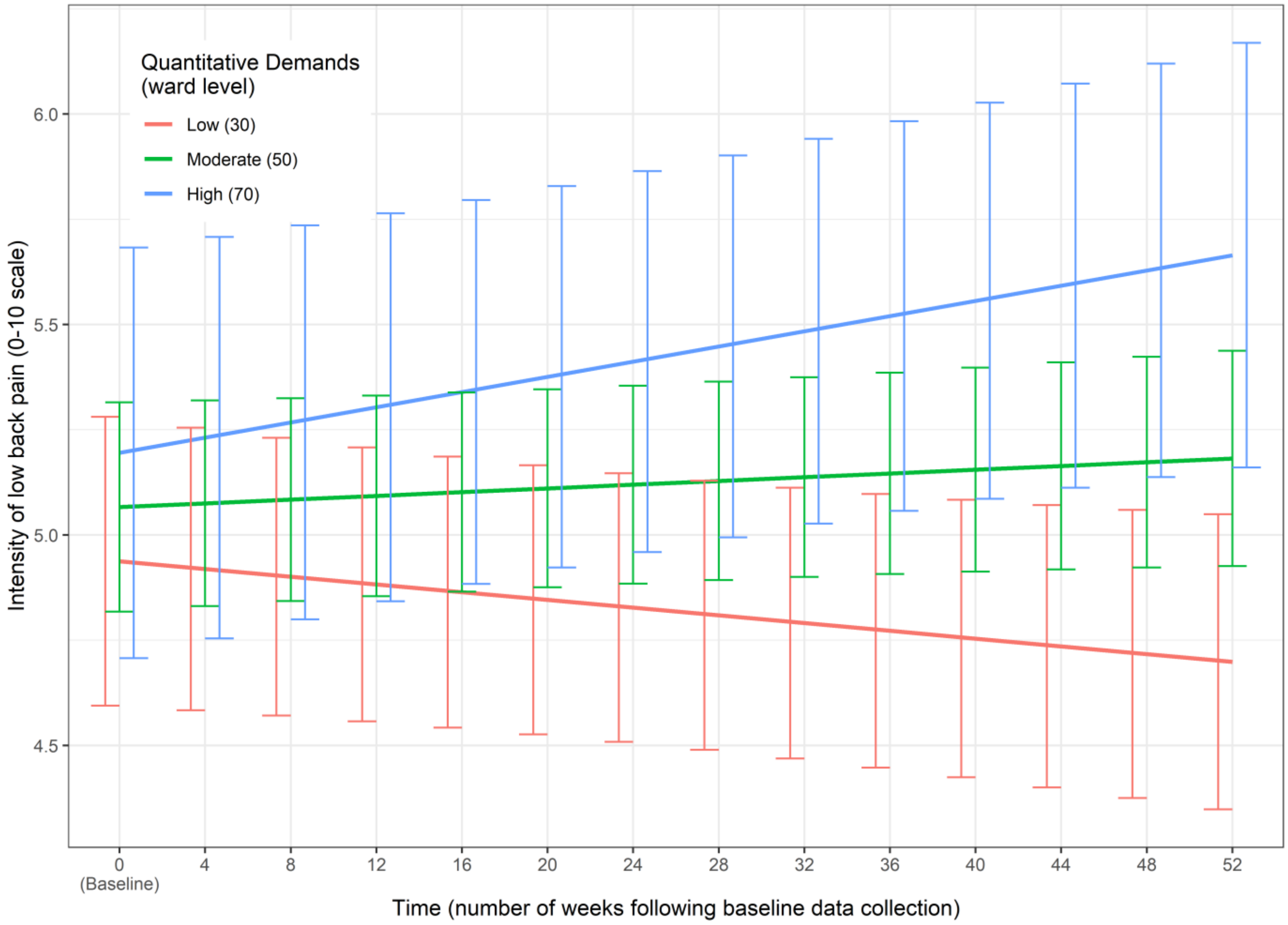

Figure S10: Interaction between Quantitative Demands (ward-level) and time on the intensity of low back pain within workers with low back pain – adjusted for age, sex, BMI emotional demands and staffing ratio

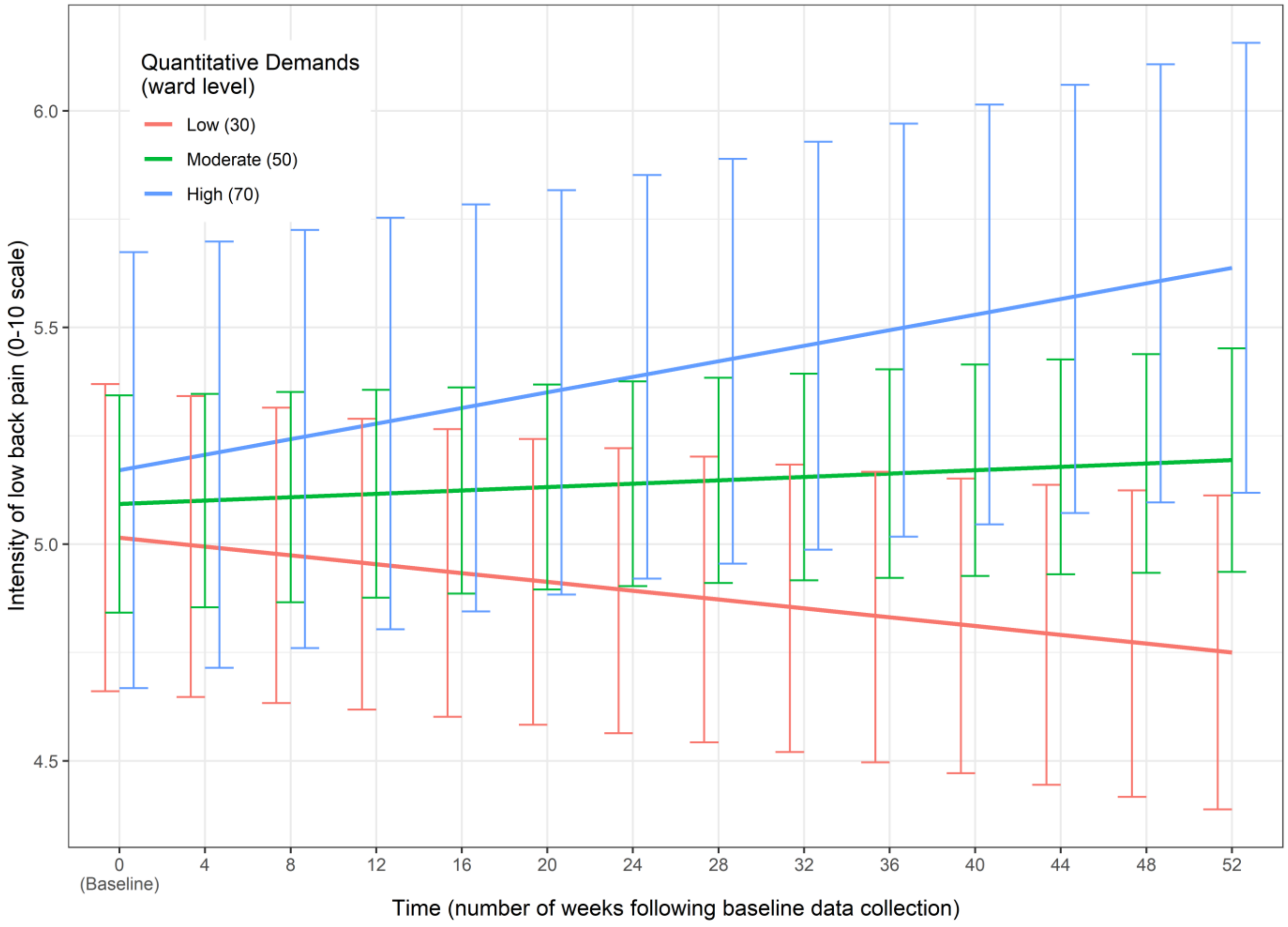

Figure S11: Interaction between Quantitative Demands (nursing home-level) and time on the intensity of low back pain within workers with low back pain – unadjusted model

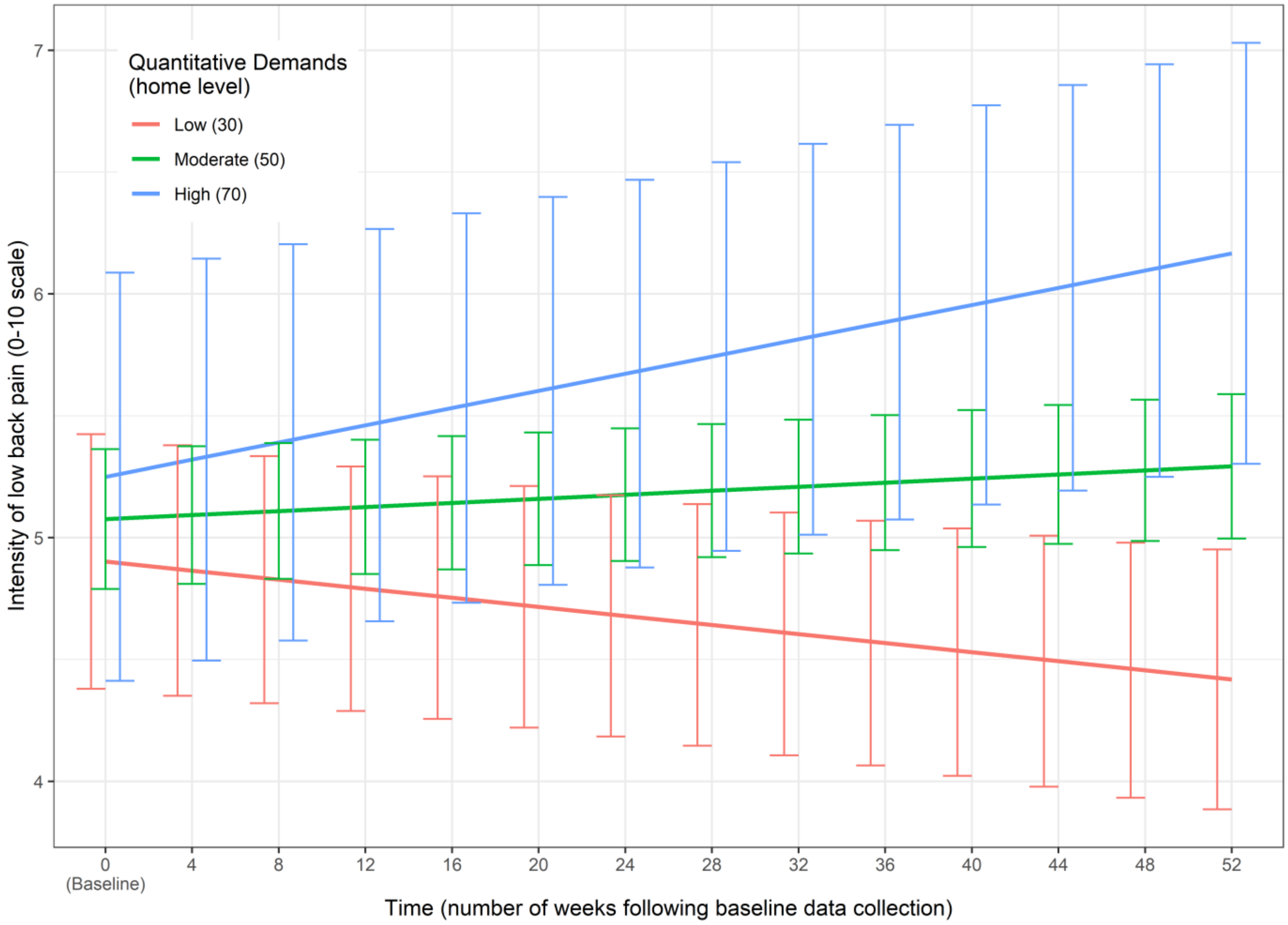

Figure S12: Interaction between Quantitative Demands (nursing home-level) and time on the intensity of low back pain within workers with low back pain – adjusted for age, sex, BMI emotional demands and staffing ratio

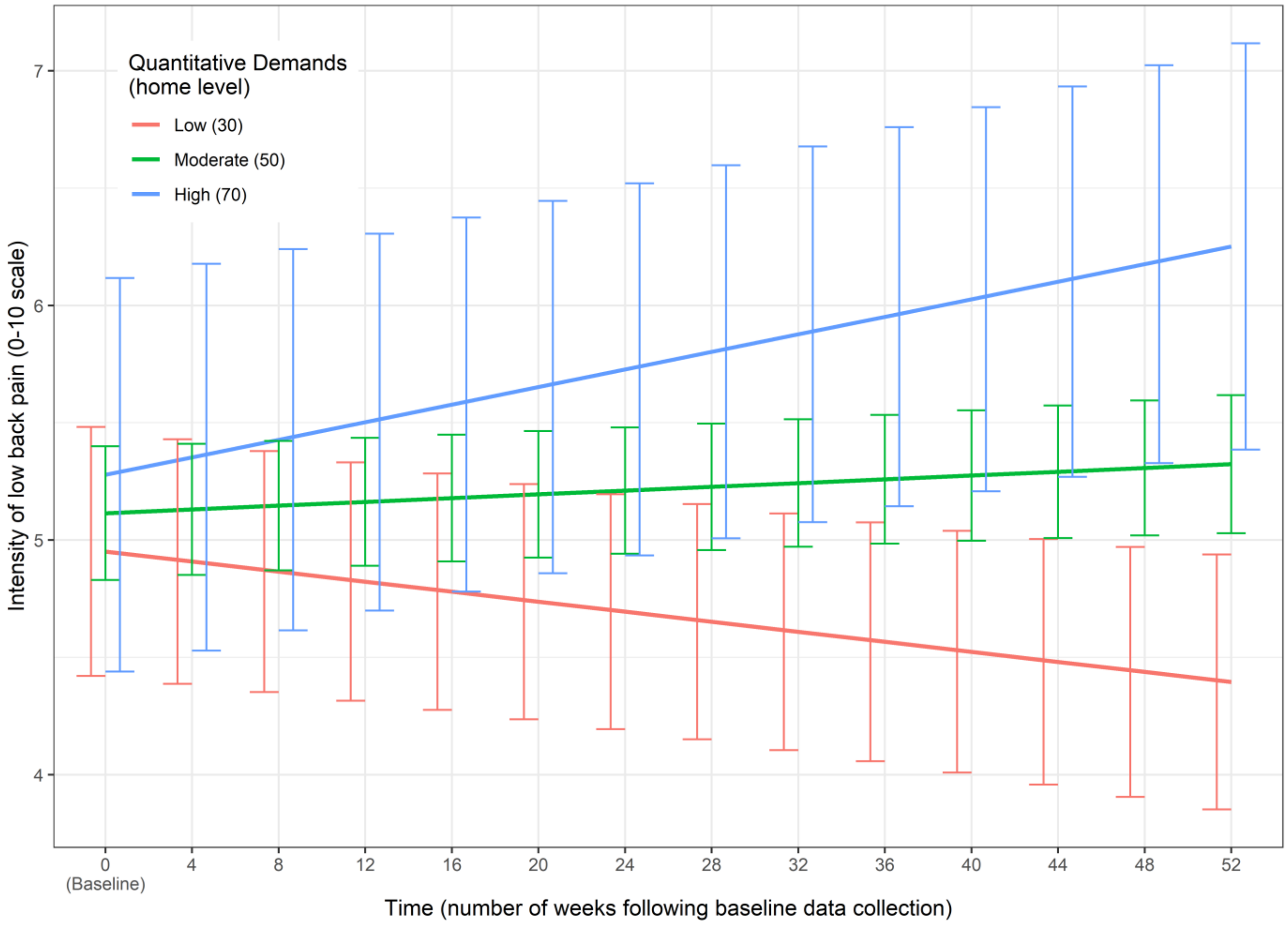

Figure S13: Interaction between Quantitative Demands (worker-level) and time on the number of days with sickness absence due to pain among workers – unadjusted model

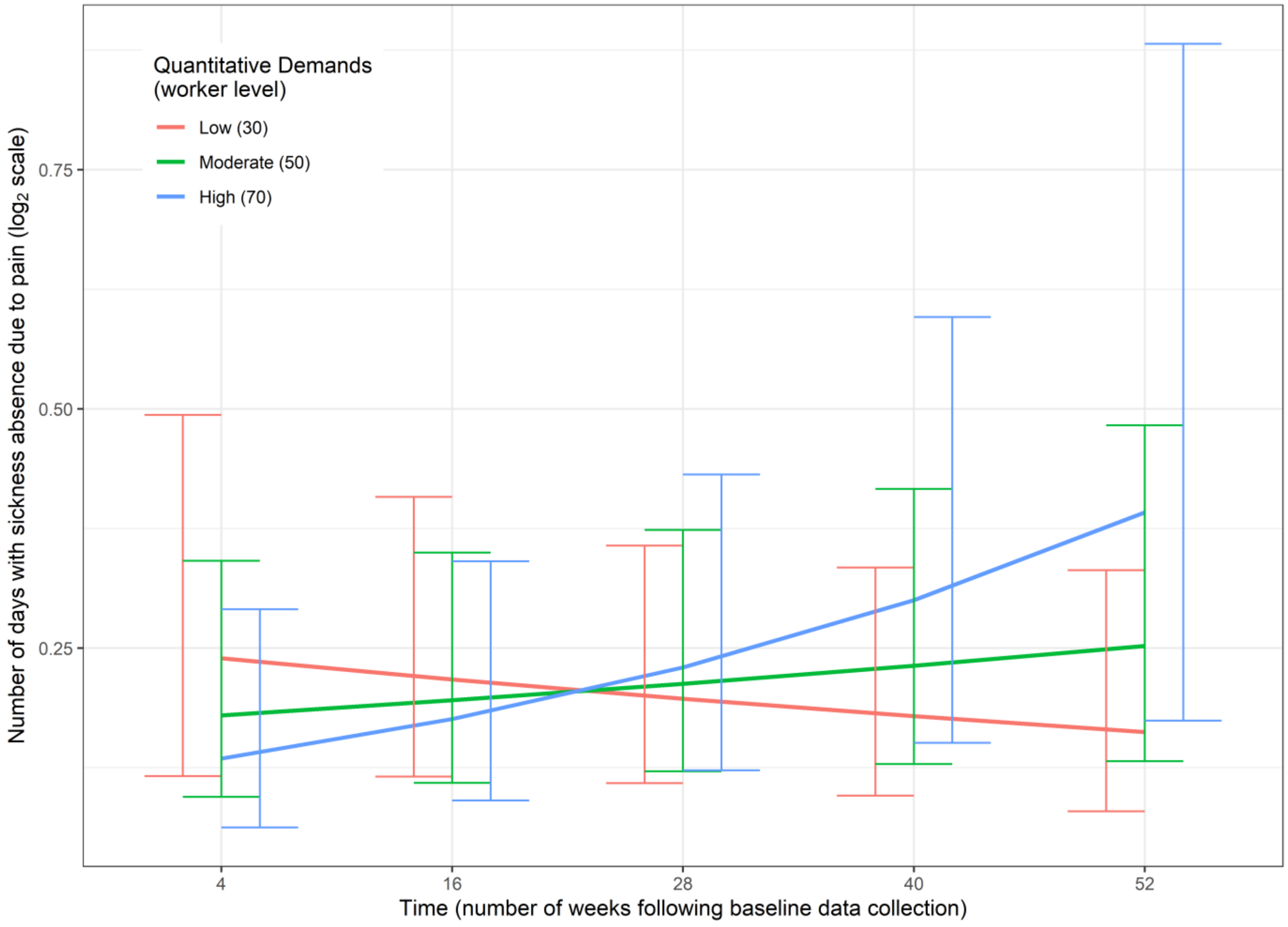

Figure S14: Interaction between Quantitative Demands (worker-level) and time on the number of days with sickness absence due to pain among workers – adjusted for age, sex, BMI emotional demands and staffing ratio

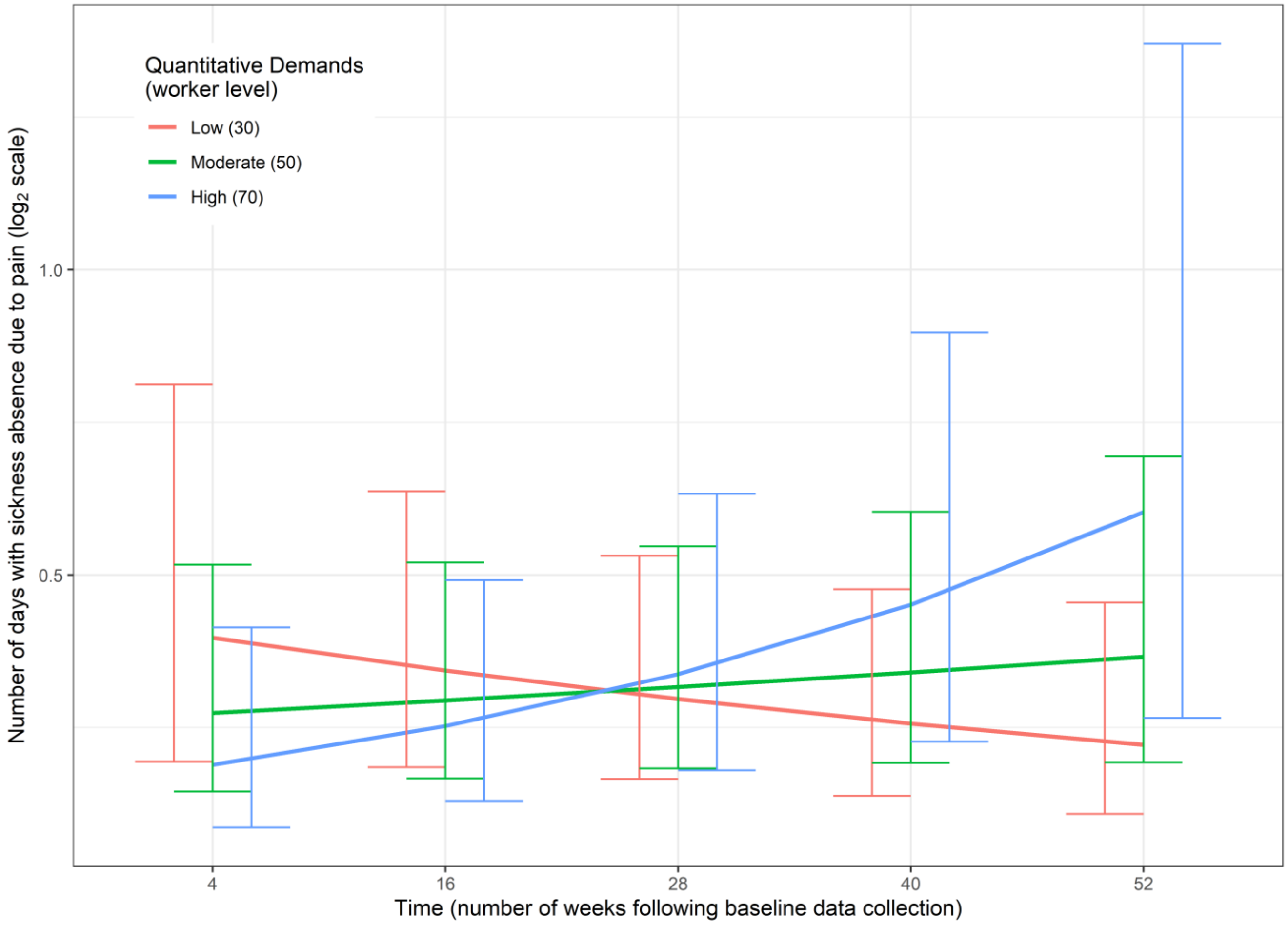

Supplement: Supplementary file 1 — Supplementary file1 (PDF 1790 kb) [file 420_2022_1850_MOESM1_ESM.pdf]
